# Anaplastic thyroid cancer cells reduce CD71 levels to increase iron overload tolerance

**DOI:** 10.1186/s12967-023-04664-9

**Published:** 2023-11-03

**Authors:** Simona D’Aprile, Simona Denaro, Anna Maria Pavone, Sebastiano Giallongo, Cesarina Giallongo, Alfio Distefano, Lucia Salvatorelli, Filippo Torrisi, Raffaella Giuffrida, Stefano Forte, Daniele Tibullo, Giovanni Li Volti, Gaetano Magro, Nunzio Vicario, Rosalba Parenti

**Affiliations:** 1 Department of Biomedical and Biotechnological Sciences, University of Catania, 95123 Catania, Italy; 2 Department of Medical and Surgical Sciences and Advanced Technologies, F. Ingrassia, University of Catania, 95123 Catania, Italy; 3 Medicine and Surgery, University of Enna “Kore”, 94100 Enna, Italy; 4 IOM Ricerca, 95029 Viagrande, Italy

**Keywords:** Thyroid cancer, CD71, Ferroptosis, Iron overload, Erastin, RSL3

## Abstract

**Background:**

Follicular thyroid cancer (FTC) is a prevalent form of differentiated thyroid cancer, whereas anaplastic thyroid cancer (ATC) represents a rare, fast-growing, undifferentiated, and highly aggressive tumor, posing significant challenges for eradication. Ferroptosis, an iron-dependent cell death mechanism driven by the excessive production of reactive oxygen species and subsequent lipid peroxidation, emerges as a promising therapeutic strategy for cancer. It has been observed that many cancer cells exhibit sensitivity to ferroptosis, while some other histotypes appear to be resistant, by counteracting the metabolic changes and oxidative stress induced by iron overload.

**Methods:**

Here we used human biopsies and in vitro approaches to analyse the effects of iron-dependent cell death. We assessed cell proliferation and viability through MTT turnover, clonogenic assays, and cytofluorimetric-assisted analysis. Lipid peroxidation assay and western blot were used to analyse molecular mechanisms underlying ferroptosis modulation. Two distinct thyroid cancer cell lines, FTC-133 (follicular) and 8505C (anaplastic), were utilized. These cell lines were exposed to ferroptosis inducers, Erastin and RSL3, while simulating an iron overload condition using ferric ammonium citrate.

**Results:**

Our evidence suggests that FTC-133 cell line, exposed to iron overload, reduced their viability and showed increased ferroptosis. In contrast, the 8505C cell line seems to better tolerate ferroptosis, responding by modulating CD71, which is involved in iron internalization and seems to have a role in resistance to iron overload and consequently in maintaining cell viability.

**Conclusions:**

The differential tolerance to ferroptosis observed in our study may hold clinical implications, particularly in addressing the unmet therapeutic needs associated with ATC treatment, where resistance to ferroptosis appears more pronounced compared to FTC.

**Supplementary Information:**

The online version contains supplementary material available at 10.1186/s12967-023-04664-9.

## Background

Thyroid cancer is the most frequent endocrine-related malignancy, representing the 5th most common cancer in women, and accounting for 3% of new incidences of all cancers per year [1–3]. Well-differentiated thyroid cancers, including papillary (PTC) and follicular (FTC), are the most common types and usually have a favourable prognosis, while undifferentiated/anaplastic carcinoma (ATC) is a less common cancer but highly aggressive, with a median survival of 2–6 months post-diagnosis [1, 4, 5]. Both types have their origins from follicular epithelial cells. However, ATC gradually develops an invasive and metastatic behaviour, while losing its follicular phenotype [4, 6–9]. Even though much progress has been made in elucidating the pathogenesis of thyroid cancers, it is still critically important to analyse the mechanisms and molecular players involved in tumorigenesis [10, 11]. In the early stages of tumor formation, indeed, follicular epithelial cells switch their phenotype from a physiological to a pathological one. These phenomena constitute the biological basis for tumor growth, cell proliferation, and therapeutic resistance.

Ferroptosis is a form of iron-dependent non-apoptotic cell death related to phospholipid peroxidation and to the release of intracellular reactive oxygen species (ROS) [12]. It is a complex process regulated by multiple cellular metabolic pathways, including redox homeostasis, iron handling, mitochondrial activity, and multiple signalling pathways [13, 14]. Several results indicate that ferroptosis holds promise as a therapeutic strategy for a number of tumors [12, 15–17]. It has been reported that ferroptosis mediators could be involved in thyroid cancer inhibition. For instance, it has been described that downregulation of solute carrier family 7 member 11 (SLC7A11), a key player for cysteine uptake in ferroptosis, inhibits PTC development [18, 19]. Furthermore, administration of vitamin C inhibits ATC development through induction of ferroptosis mediated by glutathione peroxidase 4 (GPX4) inactivation, ROS accumulation and iron-depending lipid peroxidation [20]. Moreover, it has been demonstrated that ferroptosis-related genes have a prognostic value [21–24]. As such, several mediators of ferroptosis have been identified as exploitable tools to target iron metabolism and lipid peroxidation signalling [25]. Among ferroptosis inducers, RAS-selective lethal 3 (RSL3) binds to and directly inactivates GPX4, an enzyme involved in cellular redox homeostasis, reducing membrane phospholipid hydroperoxides, thus converting toxic lipid hydroperoxides (LOOH) to non-toxic lipid alcohols (LOH) [26, 27]. Erastin is an inhibitor of SLC7A11, a subunit of the system x_c_^−^ cystine/glutamate antiporter, releasing intracellular glutamate and internalizing extracellular cystine. Erastin-mediated SLC7A11 inhibition, blocks cystine import and causes GSH depletion, leading to GPX4 enzyme inactivation [28, 29]. Ferric citrate is another ferroptosis inducer, which induces iron overload by increasing Fe^2+^ amount, leading to lipid peroxidation and consequentially ferroptosis [30–32].

Tumor cells may be able to counteract ferroptosis-related oxidative stress and metabolic alteration. Such a phenomenon may be related to their level of differentiation/dedifferentiation [13, 33, 34].

**Fig. 1:**
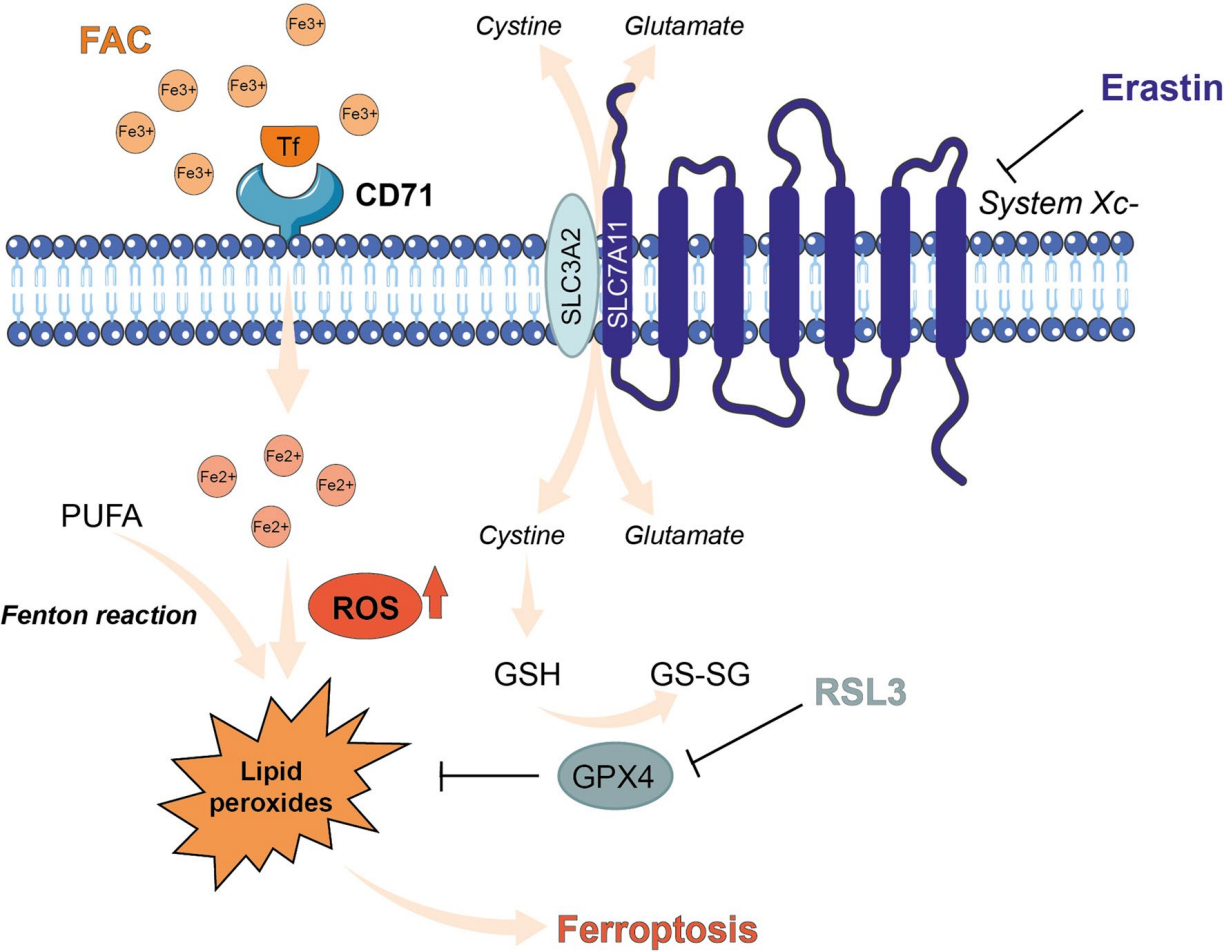
Mechanisms of ferroptotic cell death. Ferroptosis could be caused by the inhibition of system Xc− or GPX4 activity, or by iron overload which, through lipid peroxidation, lead to ferroptotic cell death. *FAC* ferric ammonium citrate, *GPX4* glutathione peroxidase 4, *GSH* glutathione, *GS-SG* glutathione disulfide, *PUFA* polyunsaturated fatty acids, *RSL3* RAS-selective lethal 3

Thus, molecular players of iron metabolism have been investigated as ferroptosis related mediators and for their role in the genesis of tumoral phenotype. Among them, type I transferrin receptor (TfR1) also known as CD71, a transmembrane glycoprotein ubiquitously present on the cell surface, is involved in iron internalization and its intracellular homeostasis (Fig. 1) [35, 36]. CD71 is overexpressed in cells with high proliferative rate, requiring more iron as a cofactor for accelerated enzymatic reactions and thus in various types of tumors including carcinomas, lymphoma, neuroendocrine and brain tumors [37–39]. CD71 is also aberrantly expressed in malignant thyroid cancers and, as we previously reported, it may represent an exploitable mediator for ATC targeted therapy [6, 40]. Recently, by studying the effects of CD71 silencing in FTC and ATC human cell lines, we have demonstrated that CD71 plays a crucial role in a rapid and transient activation of the ERK signalling pathway, which in turn induces a deregulation of genes involved in the aberrant accumulation of intracellular free iron, strongly affecting iron homeostasis in human thyroid cancer cells [41]. Moreover, the role of CD71 in mediating iron intake, has been investigated in the context of ferroptosis, finally leading to a direct association between CD71 and ferroptosis rate [42].

Herein, we exposed FTC-133 and 8505C human cell lines to ferric ammonium citrate (FAC)-mediated iron overload, Erastin or RSL3 as ferroptosis modulators, to analyse intracellular mechanisms and relationship to CD71 levels. We found that these two thyroid cancer histotypes exhibit different responses to ferroptosis stimulation, which appear to be related to CD71 level.

## Methods

### Paraffined samples of human FTC and ATC

The cases were retrospectively retrieved from the surgical pathology archives of the section of Anatomic Pathology, G.F. Ingrassia Department of Medical, Surgical, and Advanced Technologies, at University of Catania. Haematoxylin and eosin (H&E)-stained slides were available for each case. All the H&E slides were reviewed by two expert surgical pathologists and the diagnoses were histologically confirmed using the current well established morphological criteria. At least one representative formalin-fixed, paraffin embedded block, was available for immunohistochemical analyses. We selected 19 cases of minimally invasive FTC and 14 cases of ATC.

Patients with minimally invasive FTC were 8 males and 11 females, with an age ranging from 30 to 80 years (median age 52 years). Patients with ATC were 4 males and 10 females, with an age ranging from 53 to 80 years (median age 71 years).

Sections were processed as previously described [43]. Then, the sections were incubated overnight at 4 °C with rabbit polyclonal anti-CD71 antibody (Cat#136800, Invitrogen), diluted 1:200 in PBS. The secondary antibody, biotinylated anti-rabbit antibody was applied for 30 min at room temperature, followed by the avidin–biotin–peroxidase complex (Cat#PK-7100, Vector Laboratories) for a further 30 min at room temperature. The immunoreaction was visualized by incubating the sections for 4 min in a 0.1% 3,3′-diaminobenzidine (DAB) and 0.02% hydrogen peroxide solution (DAB substrate kit, Cat#SK-4100, Vector Laboratories). The sections were lightly counterstained with Mayer’s hematoxylin (Cat#01820, Histolab Products AB) mounted in GVA mountant (Zymed Laboratories) and observed with a Zeiss Axioplan light microscope (Carl Zeiss).

### Cell lines culture and drugs administration

Experiments were performed using FTC-133 and 8505C human thyroid carcinoma cell lines. Cells were purchased from European Collection of Authenticated Cell Cultures (ECACC, Public Health England). FTC-133 were cultured in Dulbecco’s Modified Eagle Medium (DMEM)/F-12 (Cat#11320033, Gibco) supplemented with 10% Foetal Serum Bovine (FBS, Cat#26140079, Gibco), 100 IU/mL Penicillin-Streptomycin solution (pen-strep, Cat#15140-122, Gibco) and 2 mmol/L l-glutamine (Cat#25030-081, Gibco); 8505C were cultured in RPMI (Cat#21875034, Gibco) supplemented with 10% FBS, 100 IU/mL pen-strep and 2 mmol/L l-glutamine. Cells were maintained in an incubator at 37 °C in a humidified atmosphere (95% air and 5% CO_2_) and were routinely sub-cultured in standard culture flasks. FAC (Cat#A11199.30, Alfa Aesar-Thermofisher) was prepared as a stock solution at 50 mM in PBS and immediately used at reported working concentrations (i.e. 5, 10, 20, 50, 100 µM). Erastin (Cat#S7242, Selleckchem) was prepared as a stock solution at 2 mM in DMSO and stored at − 20 °C. For cells treatment, drug was used at a final concentration of 10 µM. RSL3 (Cat#S8155, Selleckchem) was prepared as a stock solution at 4.53 mM in DMSO and stored at − 20 °C. For cells treatment, drug was used at a final concentration of 3 µM. Control cultures received an equal amount of vehicle (i.e. PBS and/or DMSO) as per treated cultures. In all experiments, DMSO was used at a concentration lower than 1% of the total volume.

### Metabolic turnover assay

To assess metabolic turnover of FTC-133 and 8505C cell lines, 3-(4,5-dimethylthiazol-2-yl)-2,5-diphenyltetrazolium bromide (MTT, Cat#M5655, Sigma-Aldrich) assay was performed. Cells were seeded in 96-well plates at a final density of 10000 cells/well/100 µL and incubated for 24 h. The day after, cells were exposed to increasing concentrations of FAC from 5 to 100 µM, and incubated for 24 h. MTT at a final concentration of 1 mg/mL was added to each well and incubated for 2 h and 30 min under standard culture conditions. Then, media were removed, 200 µL of MTT solvent, dimethyl sulfoxide (DMSO, Cat#P60-36720100, Pan-biotech) was added in each well, and plates were stirred on an orbital shaker for 10 min at room temperature. The absorbance was measured using a Multiskan SkyHigh Microplate spectrophotometer (Thermo Scientific) at 570 nm. Metabolic turnover was calculated as: (optical density sample/average optical density control)x100. Results were expressed as the percentage of MTT reduction versus control cells.

### Clonogenic assay

Clonogenic assay was performed by seeding cells in 6-well plates at low density: 300 cells/well for FTC-133 and 2000 cells/well for 8505C. Cells were treated with FAC at a final concentration of 50 µM and 100 µM, administrated every 48 h. After 10 days, colonies were fixed covering wells with methanol (Cat#412381, Carlo Erba) for 15 min at room temperature. Then, colonies were stained with crystal violet (Cat#61135, Sigma-Aldrich) for 25 min at room temperature. Colonies which accounted for more than 50 cells were considered as clones. Plating efficiency (P.E.) of controls was calculated as:

$$P.E.\;CTRL =\frac{number\;of \;clones}{number\; cell\; plated}.$$

The percentage of surviving fraction (S.F.) was calculated as:

$$S.F. \left(\% \;over\; CTRL\right) =\left(\frac{P.E. \;sample}{P.E. \;CTRL}\right) \times 100.$$

### Flow cytometry

For flow cytometry-assisted viability analysis, 2 × 10^5^ cells were plated in 6-well plates. The next day, cells were respectively treated with: vehicle, FAC, Erastin and RSL3. At 24 h from treatment, cells were washed and resuspended in 100 µL of PBS at 4 °C. Annexin V-FITC and propidium iodide (PI) was performed using commercially available assay kit (Cat#640922, Biolegend). Briefly, reagents were added to cell suspension and mixed gently as per manufactures’ instructions. Cells were incubated for 15 min at room temperature. Finally, 400 µL of binding buffer was added and cell preparation was analyzed by flow cytometry (MACSQuant Analyzer 10, Miltenyi Biotech). Data was evaluated using Flowlogic software (Miltenyi Biotech) [38]. ROS were detected using 2′,7′-dichlorodihydrofluorescein acetate (H2-DCF; Cat# D399, Sigma-Aldrich), and fluorescence intensity was measured according to the fluorescence detection conditions of FITC using a MACSQuant Analyzer (Miltenyi Biotech).

### Lipid peroxidation

Lipid peroxidation was evaluated by measuring levels of LOOH through a ferrous oxidation/xylenol orange assay. 1 × 10^6^ cells were plated in T25 cell plate with a culture surface of 25 cm^2^. The next day, cells were treated with RSL3 and 24 h after the treatment, cells were washed in PBS, detached using a cell culture scraper and centrifuged for 5 min at 1100×*g* to obtain dry pellet, that were stored at − 80 °C until use. To evaluate supernatant LOOH levels, cells were mechanically dissociated in 100 µL of PBS and sonicated 3 cycles for 1 min at + 4 °C. 40 µL of suspension was mixed with 200 µL of reactive mixture composed by 100 µM xylenol orange, 250 µM ammonium iron sulphate, 4 mM hydroxytoluene, 2.6 mM H_2_SO_4_ in 90% methanol. The mixture was incubated for 30 min at room temperature and then centrifugated at 11000 rpm for 5 min. The absorbance was measured at 560 nm using a Multiskan SkyHigh Microplate spectrophotometer (Thermo Scientific). Known concentrations of H_2_O_2_, from 0.2 to 20 µM, were used for calibration. The results were expressed as nmoles of LOOH/µL of supernatant.

### Western blot

For western blot analysis, cells were seeded in 6-well plates at a final density of 5 × 10^5^/well and incubated at 37 °C before drug exposure. FAC was added at the final concentration of 50 or 100 µM on cells and maintained for 24 h. Then, cells were washed in PBS, detached using a cell culture scraper and centrifuged for 5 min at 300 g to collect dry pellet, that were stored at − 80 °C until use. Proteins were extracted using RIPA Lysis Buffer (50 µL/sample; Cat#ab156034, Abcam) supplemented with protease inhibitor (1:100, Cat#P8340, Merck). Samples were incubated for 20 min at room temperature and centrifuged at 13000×*g* for 3 min. An equal amount of proteins (50 µg) were electrophoresed on 4–15% Mini-PROTEAN TGX gels (Cat#4561083, Bio-Rad) and transferred to nitrocellulose membranes, using Trans-Blot Turbo Transfer Turbo (Bio-Rad). Membranes were incubated for 1 h at room temperature with blocking buffer (5% non-fat milk in 0.1% tween-20 in PBS) and then overnight at 4 °C with primary antibodies diluted in blocking buffer. The following primary antibodies were used for western blot: mouse anti-Transferrin Receptor (1:1000, Cat#13-6800, RRID: AB_2533029, Invitrogen) and mouse β-actin (1:1000, Cat#sc-47778, RRID: AB_626632, Santa Cruz Biotechnology). Then, membranes were washed 3 times in 0.1% tween-20 in PBS and then incubated for 1 h at room temperature with the appropriate secondary antibody: Goat anti-Mouse IgG (H+L) Secondary Antibody, HRP (1:5000, Cat#31430, AB_228307, Invitrogen). Proteins bands were imaged using a ChemiDoc System (Bio-Rad) and protein levels were quantified by densitometric analysis. For immunoblotting quantification, the density of each band was quantified using ImageJ analysis software and the band density was normalized to the β-actin optical density measured in the same membrane. All values are shown as the mean fold change (FC) over control ± SEM.

### Statistical analysis

Data analysis was performed using GraphPad Prism software version 8.0.1. A two-tailed unpaired Student’s t-test was used for comparison of n = 2 groups. For comparison of n ≥ 3 groups, one-way analysis of variance (ANOVA), followed by Holm-Sidak post-hoc test for multiple comparisons were used. Data are presented as the mean ± SEM. A value of p < 0.05 was considered statistically significant and symbols used to indicate statistical differences are described in figure legends.

## Results

### Diffuse cytoplasmic staining for CD71 in FTC and ATC human sections

**Fig. 2:**
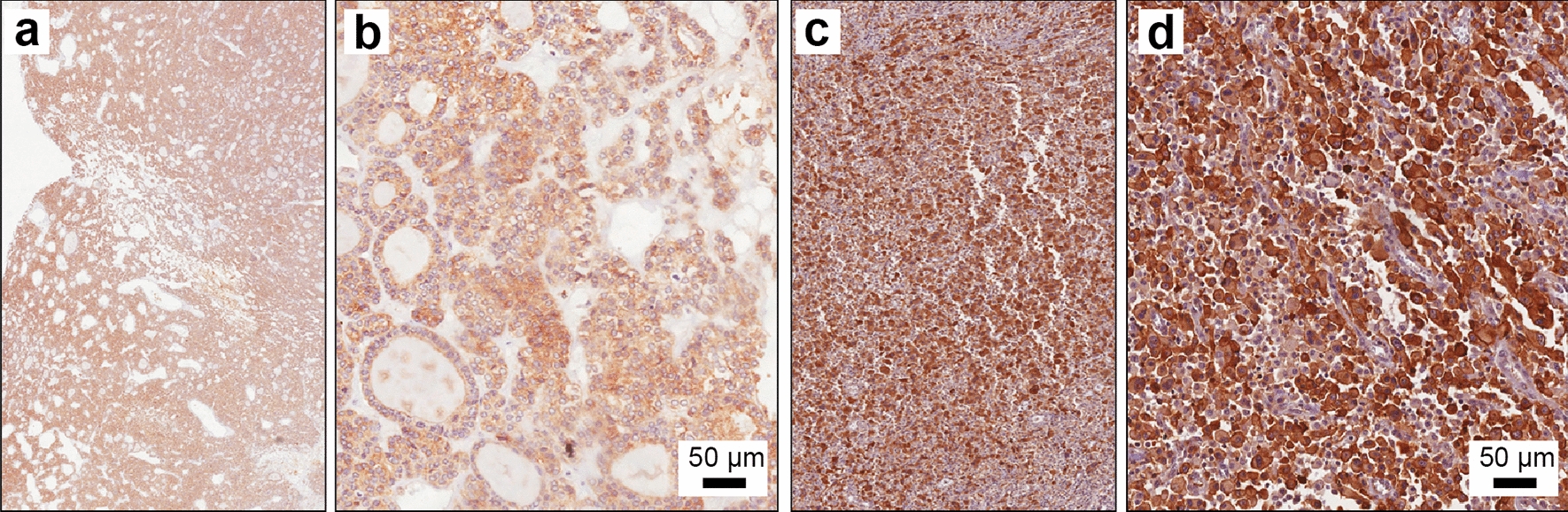
Immunohistochemical expression of CD71 on FTC and ATC human sections. **a**, **b** IHC for CD71 on human section of FTC; **c**, **d** IHC for CD71 on human section of ATC

We first analysed CD71 expression on human thyroid cancer biopsies. In minimally invasive FTC cases (19 out 19), a diffuse and moderate cytoplasmic staining of CD71 was observed (Fig. 2a, b). In all tested ATCs cases (14 out 14) we observed an overexpression of CD71 as compared to FTC, along with a diffuse and strong cytoplasmic and membrane staining (Fig. 2c, d).

### Iron overload induces early responses in FTC-133 but not in 8505C cells

**Fig. 3:**
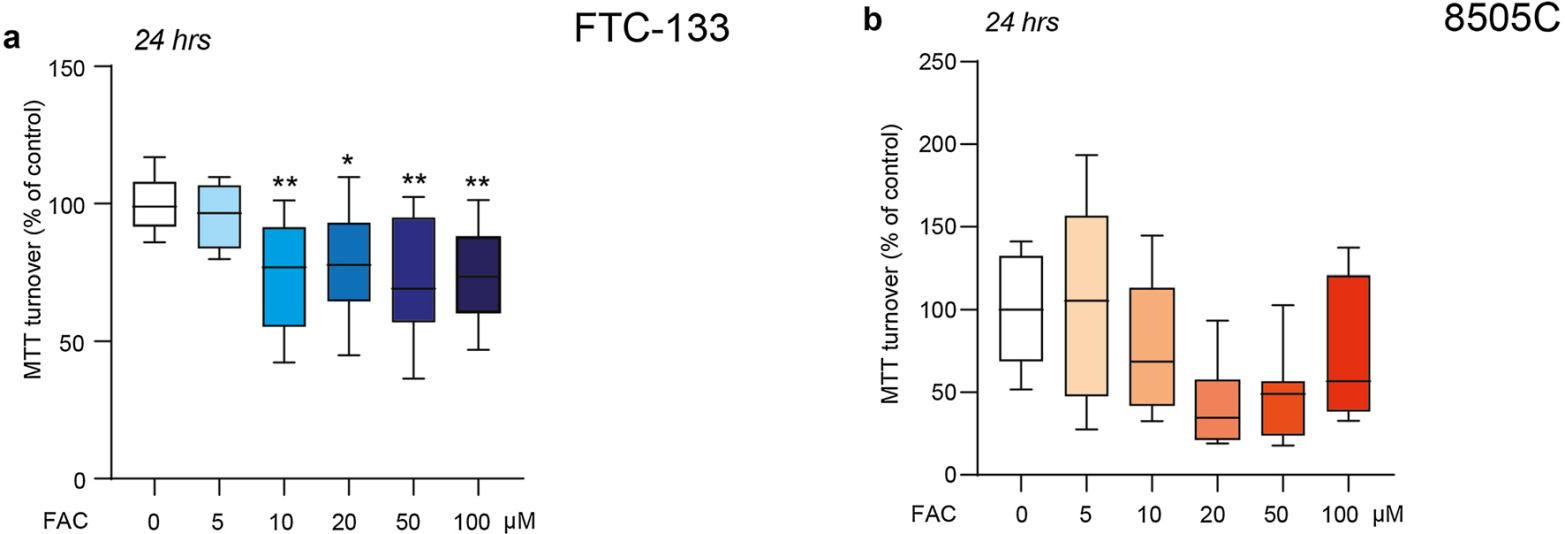
FAC reduces metabolic turnover in FTC-133 but not in 8505C cells. **a**, **b** MTT turnover on FTC-133 (**a**) and 8505C (**b**) treated with 0, 5, 10, 20, 50, 100 µM of FAC for 24 h. Data are shown via standard box and whiskers of n ≥ 4 independent replicates for each experimental condition. *p-value < 0.05; **p-value < 0.01 versus 0 µM controls. FAC, ferric ammonium citrate

We evaluated the effects of FAC on metabolic turnover mimicking an intracellular condition of iron overload by exposing both analysed cell lines, FTC-133 and 8505C, to increasing FAC concentrations (5, 10, 20, 50, 100 µM). Metabolic turnover was evaluated by MTT assay 24 h after treatment. A different response between tested cell lines was observed, in particular FAC induced an early reduction of MTT metabolic turnover in FTC-133 cells starting from 10 µM FAC concentration up to 100 µM (73.95% ± 5.60% for 10 µM FAC, p-value = 0.0061; 77.92% ± 5.19% for 20 µM FAC, p-value = 0.0312; 72.77% ± 6.24% for 50 µM FAC, p-value = 0.0041; 73.15% ± 4.76% for 100 µM FAC, p-value = 0.0046; versus control: 100.0% ± 2.62%). On the contrary, 8505C cells exposed to FAC did not show any significant change at all tested concentrations (Fig. 3).

**Fig. 4:**
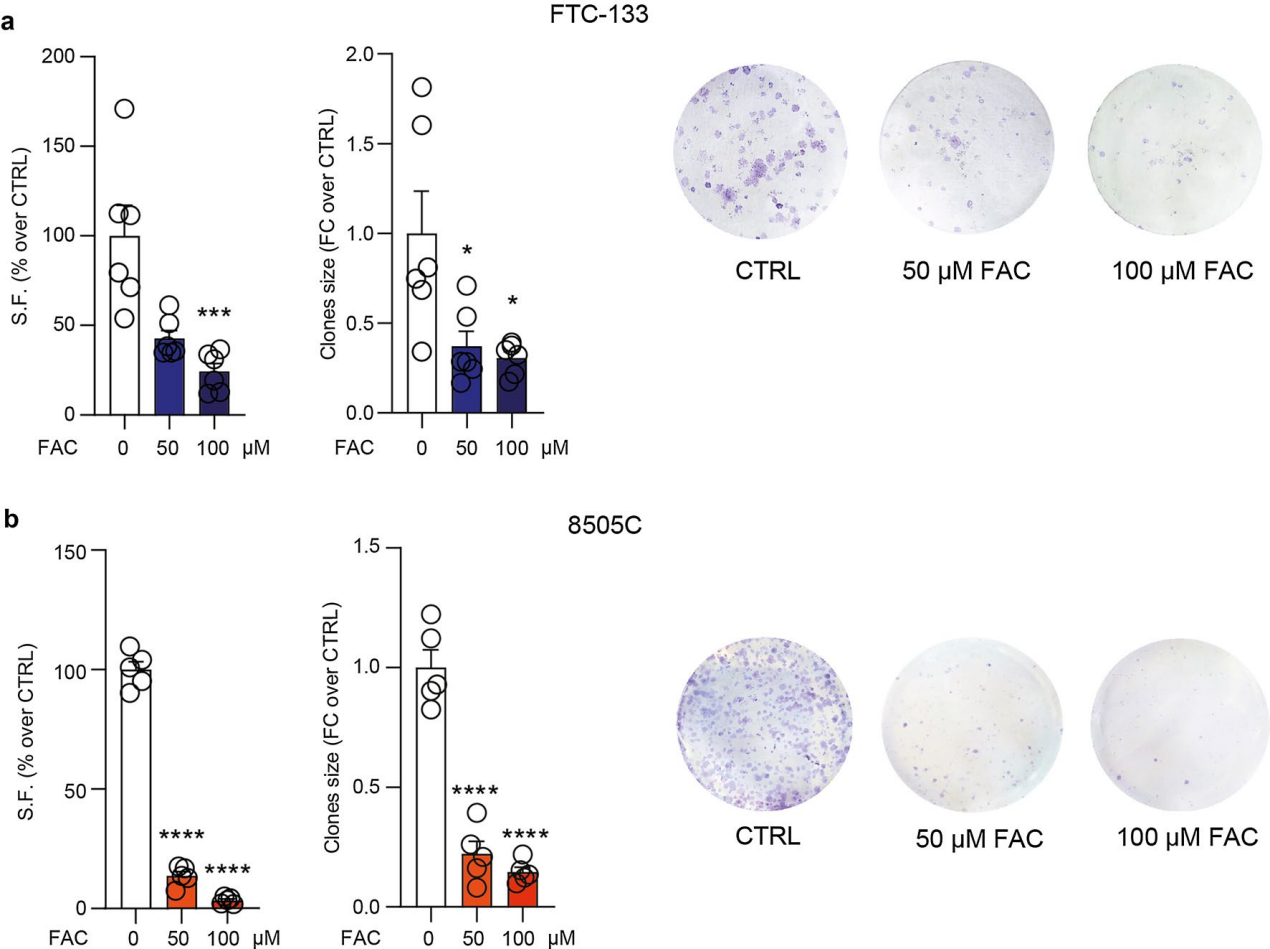
FAC decreases FTC-133 and 8505C clonogenicity. **a**, **b** S.F. (% over CTRL), clones size (FC over CTRL) and representative pictures of FTC-133 (**a**) and of 8505C (**b**) treated with 0, 50, 100 µM of FAC. Data are expressed as mean ± SEM of n ≥ 5 independent experiments. *p-value < 0.05; ***p-value < 0.001 and ****p-value < 0.0001 versus 0 µM controls. *S.F.* surviving fraction, *FC* fold change, *CTRL* control, *FAC* ferric ammonium citrate

We then moved to analyse the chronic effect of FAC treatment on FTC-133 and 8505C clonogenicity, by administering FAC every 48 h. Our results showed that both cell lines respond to FAC treatment reducing the S.F. in FTC-133 cells (42.68 ± 4.52, p-value = 0.1359 for 50 µM FAC-treated FTC-133; 24.24 ± 4.47, p-value = 0.0007 for 100 µM FAC-treated FTC-133 versus FTC-133 control: 100.0 ± 17.02, Additional file 1: Table S1 and Fig. 4a) and in 8505C cells (13.66 ± 1.80 for 50 µM FAC-treated 8505C, p-value < 0.0001; 3.36 ± 0.61, p-value < 0.0001 for 100 µM FAC-treated 8505C; versus 8505C control: 100.0 ± 3.39, Additional file 1: Table S1 and Fig. 4b). A significant reduction was observed also in clone size of either FTC-133 cells (0.37 ± 0.08, p-value = 0.0162 for 50 µM FAC-treated FTC-133; 0.30 ± 0.04, p-value = 0.0126 for 100 µM FAC-treated FTC-133; versus FTC-133 control: 1.00 ± 0.24, Fig. 4a) and 8505C cells (0.22 ± 0.05 for 50 µM FAC-treated 8505C, p-value < 0.0001; 0.15 ± 0.02, p-value < 0.0001 for 100 µM FAC-treated 8505C; versus 8505C control: 1.00 ± 0.07, Fig. 4b). Collectively, this evidence indicates that FTC-133 cells show a rapid response to FAC treatment in terms of metabolic turnover. However, both FTC-133 and 8505C cells exhibit similar proliferation rates in response to long-term FAC exposure.

### Erastin induces oxidative damage in FTC-133 cells

**Fig. 5:**
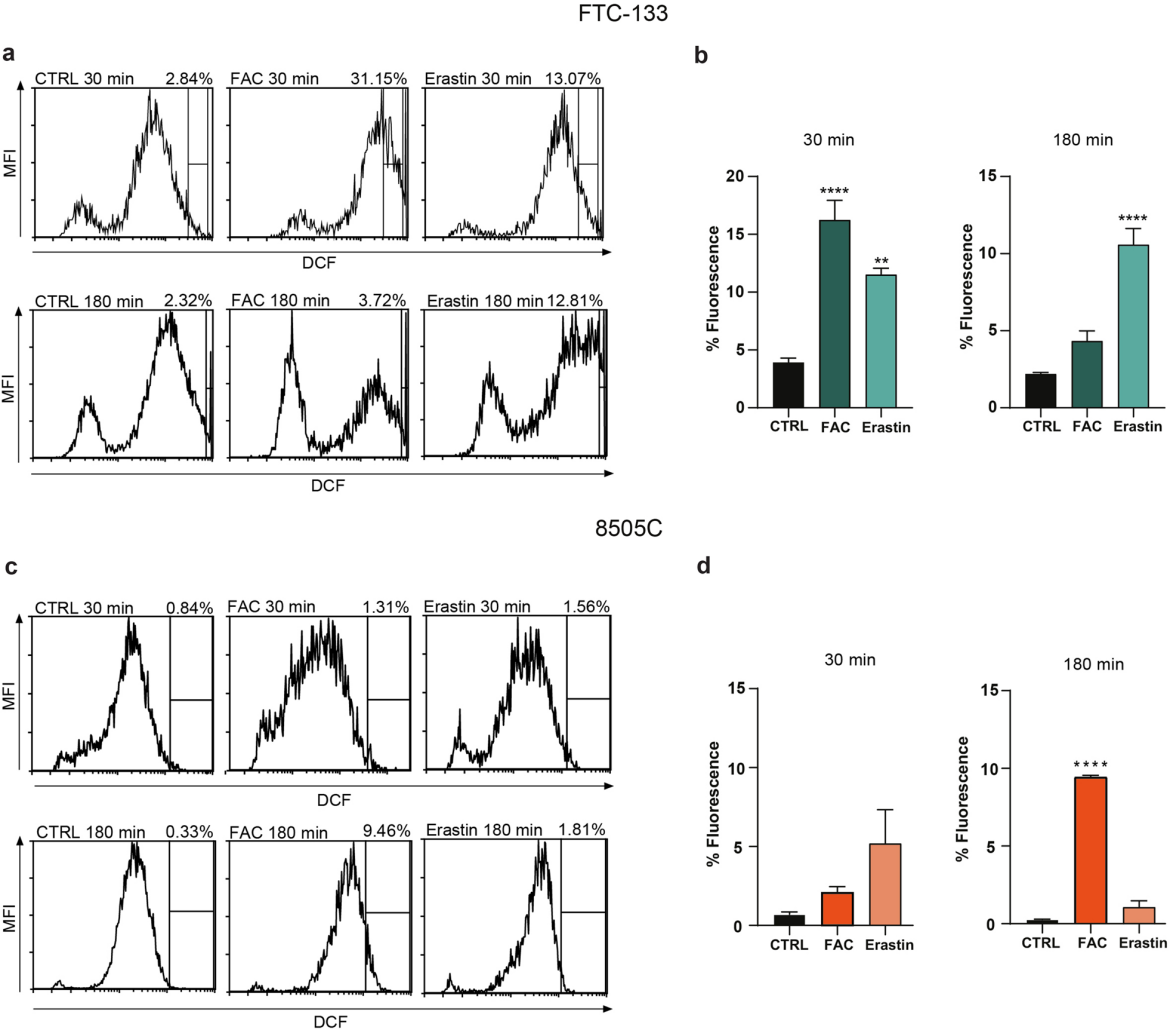
Erastin leads to ROS overproduction in FTC-133 but not in 8505C. **a**–**d** Representative plots of ROS production in FTC-133 (**a**) and in 8505C (**c**), using DCF in flow cytometry, following 30 min and 180 min of FAC and Erastin treatment. Percent fluorescence quantification of DCF for FTC-133 (**b**) and for 8505C (**d**) is expressed in the two time-points. Data are shown as mean ± SEM of n = 4 independent experiments. **p-value < 0.01 and ****p-value < 0.0001. *CTRL* control, *FAC* ferric ammonium citrate, *MFI* mean fluorescence intensity, *DCF* 2′,7′-dichlorodihydrofluorescein acetate

Since high levels of iron are responsible for ROS overproduction, we evaluated the amount of intracellular ROS, 30 and 180 min after ferroptosis induced by either FAC or Erastin administration. We observed ROS overproduction in FTC-133 cell line at 30 min after FAC administration (16.25% ± 1.70%, p-value < 0.0001 for FAC-treated FTC-133 versus FTC-133 control: 3.91% ± 0.40%, Fig. 5a, b), while 8505C cells showed an increase in ROS levels after 180 min of FAC treatment (9.43% ± 0.13%, p-value < 0.0001 for FAC-treated 8505C versus 8505C control: 0.27% ± 0.02%, Fig. 5c, d). On the other hand, we observed a significant ROS increase in FTC-133 cell line both at 30 min (11.53% ± 0.55%, p-value = 0.0013 versus FTC-133) and 180 min following Erastin administration (10.58% ± 1.06%, p-value < 0.0001 versus FTC-133 control, Fig. 5a, b). While no significant changes were found in the amount of ROS in 8505C cells exposed to Erastin at the tested time-points (Fig. 5c, d).

**Fig. 6:**
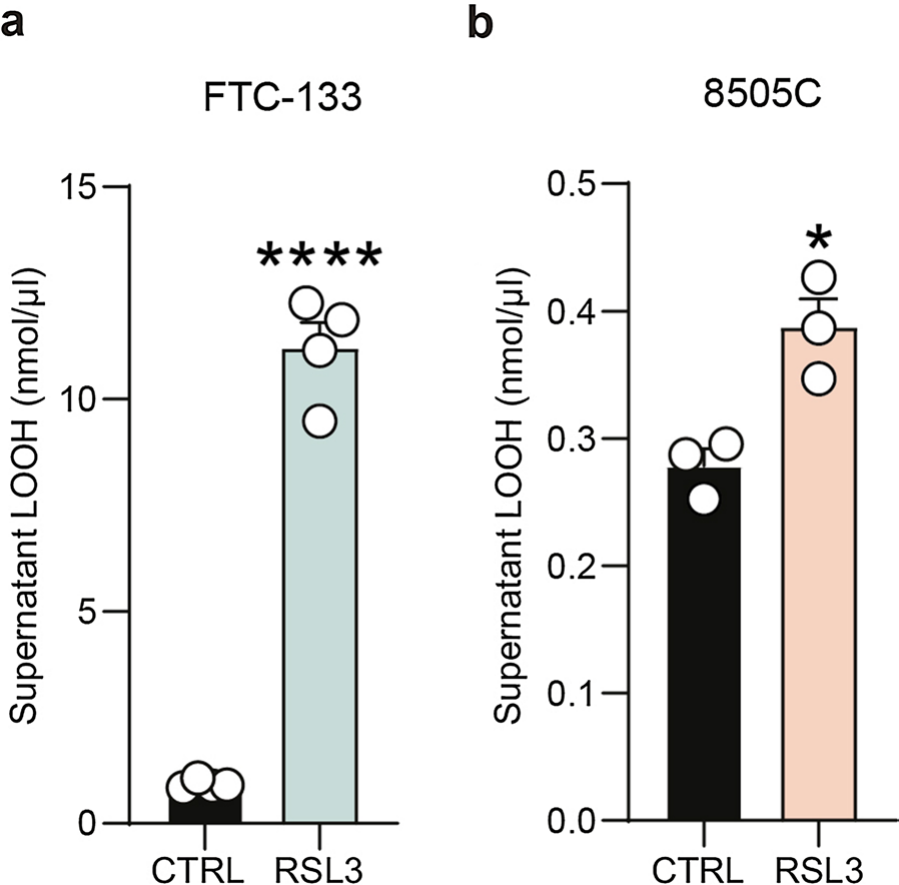
RSL3 strongly increases LOOH production in FTC-133. **a** Supernatant LOOH (nmol/µL) in FTC-133, 24 h after RSL3 treatment. **b** Supernatant LOOH (nmol/µL) in 8505C, 24 h after RSL3 treatment. Data are shown as mean ± SEM of n ≥ 3 independent experiments. *p-value < 0.05 and ****p-value < 0.0001 versus control. *CTRL* control, *RSL3* RAS-selective lethal 3

Afterwards, we analysed the effects of RSL3, an inhibitor of the enzyme GPX4 converting LOOH to LOH, on both cell lines. LOOH has been assessed in order to analyse lipid peroxidation after treatment. Our data showed that RSL3 increased LOOH levels after 24 h in both tested cell lines (Fig. 6a, b). It is worth noticing that we observed an increase of about 11 folds in FTC-133 LOOH as compared to controls (11.19 ± 0.61, p-value < 0.0001 for RSL3-treated FTC-133 versus FTC-133 control: 0.92 ± 0.05, Fig. 6a), whereas in 8505C cell lines the LOOH levels were only slightly augmented (0.39 ± 0.02, p-value = 0.0150 for RSL3-treated 8505C versus 8505C control: 0.28 ± 0.01, Fig. 6b). This finding suggests that FTC cells are more sensitive to lipid peroxidation induced by GPX4 inhibition as compared to 8505C cells.

### 8505C cells decrease CD71 expression levels to resist to ferroptosis

**Fig. 7:**
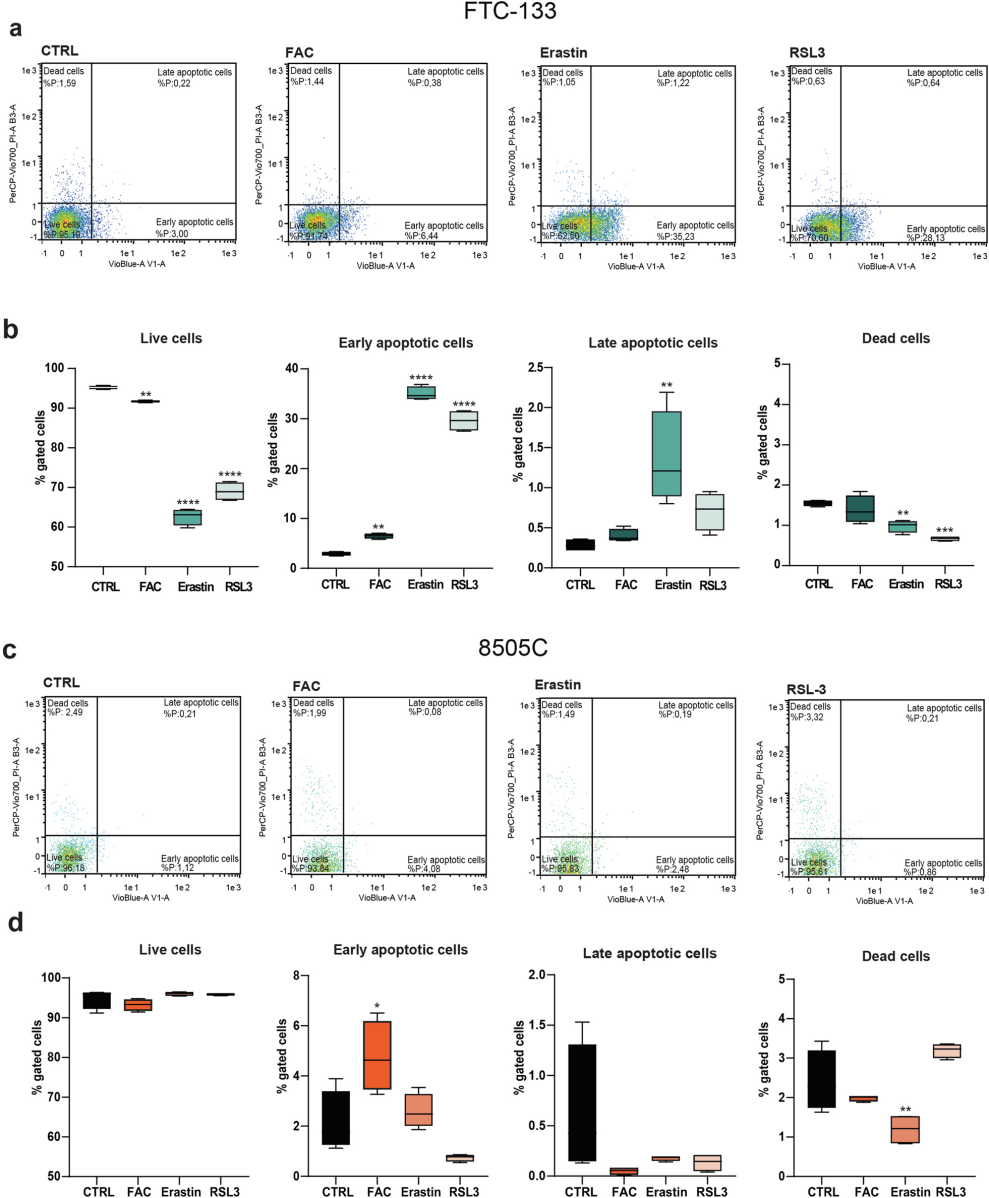
Ferroptosis activators and iron overload reduce FTC-133 viability, but do not affect 8505C viable cells. **a**–**d** Cytofluorimetric analysis of viability evaluated with Annexin V/PI assay on FTC-133 treated with FAC, Erastin and RSL3 (**a**, **b**) and on 8505C treated with FAC, Erastin and RSL3 (**c**, **d**). Data are shown as standard box and whiskers and viability is expressed as percentage of gated cells, n = 4 independent replicates for each experimental condition. *p-value < 0.05; **p-value < 0.01; ***p-value < 0.001; ****p-value < 0.0001 versus control. *CTRL* control, *FAC* ferric ammonium citrate, *RSL3* RAS-selective lethal 3

To assess cell resistance to ferroptosis induction, we exposed FTC-133 and 8505C cell lines to FAC, Erastin or RSL3 for 24 h, and then we analysed the effects on cell viability, apoptosis and cell death via Annexin V/PI assay. Our data showed that FTC-133 viability was significantly affected by all the three ferroptosis inducers (91.72% ± 0.13%, p-value = 0.0086 for FAC-treated FTC-133; 62.63% ± 1.03%, p-value < 0.0001 for Erastin-treated FTC-133; 69.03% ± 1.18%, p-value < 0.0001 for RSL3-treated FTC-133; versus FTC-133 control: 95.23% ± 0.20%, Fig. 7a, b). These data were coupled with a significant increase in the proportion of early apoptotic cells (6.49% ± 0.26%, p-value = 0.0020 for FAC-treated FTC-133; 35.04% ± 0.68%, p-value < 0.0001 for Erastin-treated FTC-133; 29.60% ± 1.03%, p-value < 0.0001 for RSL3-treated FTC-133; versus FTC-133 control: 2.94% ± 0.18%, Fig. 7a, b). Notably, ferroptosis modulators showed different effects on 8505C cells. Our data suggested that none of the tested modulators affected cell viability, with just slight changes in the proportion of early apoptotic cells upon FAC administration (4.76% ± 0.70%, p-value = 0.0143 for FAC-treated 8505C versus 8505C control: 2.15% ± 0.60%), but no evident modifications were seen in the proportion of viable cells (Fig. 7c, d).

Our data demonstrated that 8505C cell lines better tolerate the effects mediated by ferroptosis inducers, maintaining their viability following administration of FAC, Erastin, and RSL3. On the contrary, FTC-133 cells show a much higher sensitivity to ferroptosis inducers, showing a significant reduction of their viability and a contemporary increase in the amount of early apoptotic cells following all three different treatments.

**Fig. 8:**
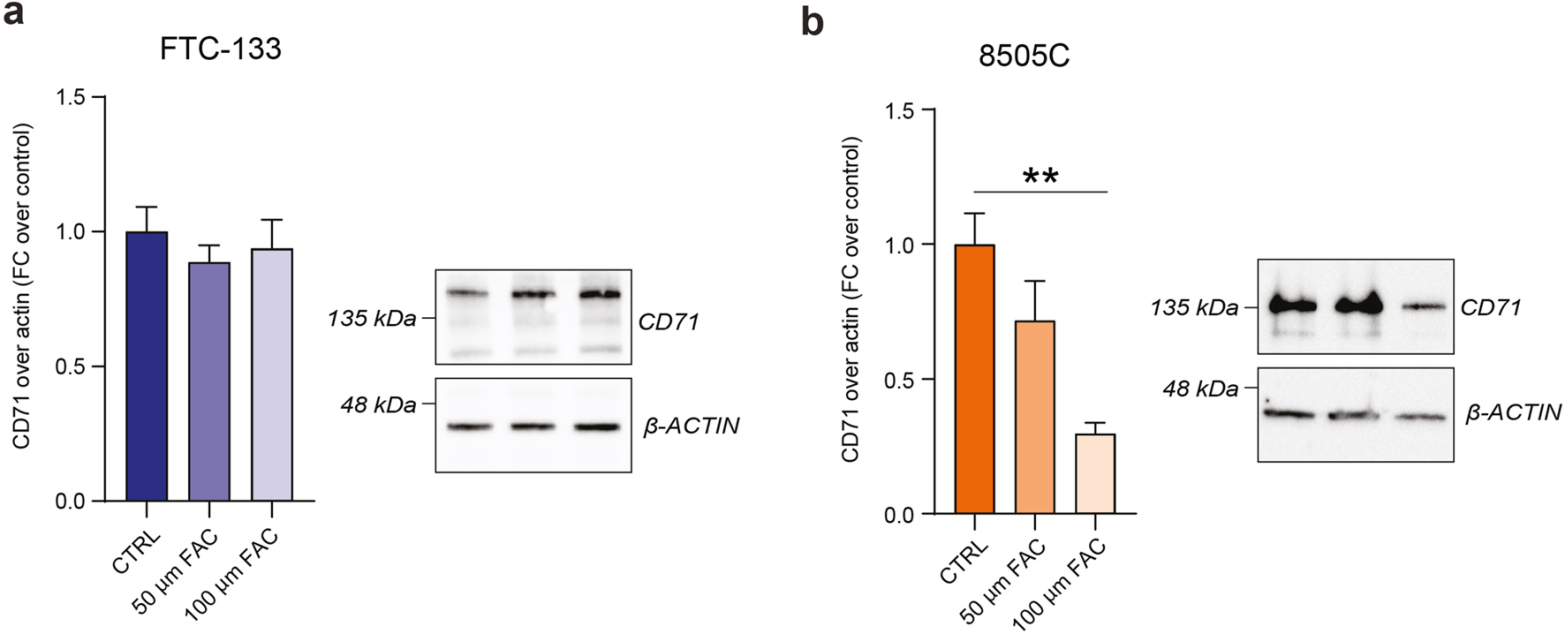
8505C cells reduce CD71 expression levels to tolerate ferroptosis. **a**, **b** Western blot analysis of CD71 expression levels and representative cropped blots on FTC-133 cells (**a**) and 8505C (**b**) exposed to 50 or 100 µM of FAC. Data are mean FC over control ± SEM of n ≥ 3 independent experiments. **p-value < 0.01. *CTRL* control, *FAC* ferric ammonium citrate, *FC* fold change

Finally, to better elucidate the potential role of CD71 in cell resistance to ferroptosis and its possible involvement in iron overload regulatory process, we tested CD71 expression levels in both cell lines upon FAC administration. As shown in Fig. 8 and in Additional file 1: Fig. S1, FTC-133 cells did not show any significant variation of CD71 expression levels following FAC exposition, whereas 8505C cells displayed a significant reduction of CD71 expression levels when exposed to 100 µM FAC (0.30 ± 0.04, p-value < 0.01 for 100 µM FAC-treated 8505C versus control: 1.00 ± 0.11). These data suggest that 8505C cells modulate CD71 levels to increase iron-overload tolerance, while less aggressive FTC-133 cells are unable to reshape their phenotype upon FAC exposure.

## Discussion

Thyroid cancer is the most prevalent endocrine malignancy worldwide, and its incidence is rapidly rising. A portion of this increase may be attributed to advancements in diagnostic techniques, such as high-resolution imaging and molecular assays, enabling the identification of previously undetectable thyroid cancer formations [44, 45]. Differentiated thyroid cancers retain some biological characteristics of normal thyroid cells, including the ability to absorb and process iodine, allowing the use of radioactive iodine for scintigraphy and conventional therapy [45]. In contrast, undifferentiated thyroid cancers, such as ATC, are extremely aggressive, early metastatic and resistant to conventional therapeutic strategies [45, 46]. Thus, new effective therapies for ATC represent an unmet clinical need, particularly in advanced or metastatic stages, which are still approached via palliative care [7].

Several data supported ferroptosis as a promising approach for cancer therapy [15, 47]. On the one hand, cancer cells strongly increase their iron demand to support the high proliferative rate and tumor progression; on the other hand, the increase in free intracellular iron is able to trigger metabolic alterations, lipid peroxidation and enzyme modifications, inducing the so-called ferroptosis [48, 49]. However, in some cases, this therapeutic strategy has been hampered by resistance mechanisms [13, 50, 51]. Indeed, cancer cells can adapt their metabolism to the oxidative environment, also reshaping tumor niches, triggering oncogenic mutations, thus enabling cancer growth and progression [52, 53]. In recent years numerous studies have explored the cellular processes involved in ferroptosis tolerance. However, the mechanisms underlying resistance to this process remain largely unknown and uncharacterized and new research tools are needed to investigate tumor biology and therapeutic opportunity [54, 55].

Our previous study demonstrated a critical role of CD71, becoming part of an aberrant gene/protein expression pattern upon neoplastic transformation and malignant progression of ATC, which strongly candidate it as potential direct or indirect therapeutic target [40]. In particular, the core of its therapeutic potential could be also the close relationship with iron-depending cell death [56]. For instance, CD71 is mainly involved in iron homeostasis, binding serum transferrin and thus contributing to the increase of the small pool of Fe^2+^ (referred to as the labile iron pool), able to generate ROS, which in turn initiate lipid peroxidation and eventually lead to ferroptosis [15, 57]. Compelling evidence suggest that CD71 RNAi leads to a significant decrease in cell death, implying its involvement in ferroptosis via cell metabolism and redox machinery [58]. Thus, CD71 reduction may represent a protective cellular process to overcome iron overload-induced damages. Indeed, the proteasome inhibitor Bortezomib, a compound commonly used in the setting of multiple myeloma patients, impairs the physiological defensive response to iron overload that consists in CD71 degradation and ferritin increase, thus maximizing the toxicity of supplemented iron and leading to ferroptosis-mediated cell death [14, 59–61].

Here, we investigated the effects of iron overload by administering FAC to FTC-133 and 8505C cell lines, which represent models of differentiated and undifferentiated/ATC, respectively. This condition was observed to decrease cell viability and to increase intracellular ROS levels, finally leading to iron overload-induced cell death [62–64]. We evaluated the effects of FAC on cellular metabolic turnover at 24 h post-treatment. Our data showed that 10 µM concentration of FAC was sufficient to reduce metabolic turnover in FTC-133 cells. Conversely, 8505C cells did not show any significant changes following a single-dose FAC treatment, at all tested concentrations. However, we observed a similar reduction in clonogenicity, expressed as S.F. and size of clones, in both cell lines exposed to repeated administration of FAC. Afterwards, we tested different conditions of ferroptosis perturbation by investigating the effects following to Erastin and RSL3 treatments. Erastin has been shown to be effective in various cancer types, exhibiting a synergistic effect when used in combination with others chemotherapeutic drugs [65, 66]. However, it has been observed that in some cancer conditions, treatment with Erastin could induce ferroptosis resistance [67]. Indeed, Erastin enhances metastatic ability of ferroptosis-resistant ovarian cancer cells via macrophages M2-like polarization, through STAT3 pathway and IL-8 release [68].

We analysed intracellular ROS accumulation following either FAC or Erastin administration. By flow cytometry-assisted analysis, we observed that 8505C cells showed high tolerance to ferroptosis inducers, near-normal levels of cell viability and limited ROS overproduction at all tested timepoints.

In contrast, FTC-133 cells response was characterized by a decrease in the number of viable cells and a concomitant increase in apoptotic cells, along with ROS accumulation, confirmed in both the early and late stages of treatment.

Although the mechanisms underlying ferroptosis are not fully clarified, RSL3 has been used as a potent ferroptosis triggering agent in many types of cancer, inhibiting GPX4 activity [69, 70].

Our data showed that RSL3 treatment induces lipid peroxidation in both FTC and ATC cell lines, with an increase in LOOH levels. Such an increase was much more pronounced in FTC-133 than in 8505C. Furthermore, RSL3-treated FTC-133 cells showed a significant reduction in the proportion of viable cells, while there were no significant changes in cell viability in 8505C cells. Finally, we investigated CD71 levels in FTC-133 and 8505C cells in iron overload conditions. We did not find any significant changes in CD71 levels in FTC-133 cells exposed to FAC, while conversely, a decrease in CD71 expression was observed in 8505C cells. This outcome suggests the activation of a protective mechanism in the ATC cell line, involving the modulation of CD71 levels. We hypothesize that iron overload activates ferroptosis in FTC cells, which are not able to counteract iron-mediated cell death. Vice versa, ATC cells are able to develop resistance to ferroptosis, thus tolerating iron overload-induced toxicity, via CD71 modulation and oxidative rebalancing.

## Conclusions

Our results suggest that thyroid cancers are differently responsive to iron overload. FTC-133 cells are sensitive to iron metabolism alteration, which induces ROS overproduction, increased lipid peroxidation and ferroptosis activation. This suggests iron metabolism as an exploitable therapeutic target for differentiated thyroid cancers. On the contrary, 8505C cells are resistant to iron overload, but also to the mechanisms leading to ferroptosis induced by specific activators, such as Erastin and RSL3. CD71 appears to be involved in these mechanisms by modulating iron intracellular levels.

In conclusion, our results demonstrate ability of ATC to better tolerate extreme conditions, nevertheless the analysis of the crosstalk between the molecular players of its ferroptosis resistance shows the involvement of CD71 modulation, which represents an early player in the iron handling process. As such, acting on CD71 regulation could provide an Achilles’ heel to defeat such an aggressive tumor and shade lights on this dismal disease.

### Supplementary Information

**Additional file 1: Table S1.** Descriptive statistics for the S.F. (% over CTRL) observed in FTC-133 and 8505C cells, after treatment with 0, 50, 100 μM of FAC. S.F., surviving fraction; CTRL, control; FAC, ferric ammonium citrate. **Figure S1.** Original, uncropped western blot images showed in Figure 8 for FTC-133 (a) and for 8505C (b). Samples are indicated as a1 = CD71 FTC-133 CTRL; a2 = CD71 FTC-133 50 μm FAC; a3 = CD71 FTC-133 100 μm FAC; a4 = β-ACTIN FTC-133 CTRL; a5 = β-ACTIN FTC-133 50 μm FAC; a6 = β-ACTIN FTC-133 100 μm FAC; b1 = CD71 8505C CTRL; b2 = CD71 8505C 50 μm FAC; b3 = CD71 8505C 100 μm FAC; b4 = β-ACTIN 8505C CTRL; b5 = β-ACTIN 8505C 50 μm FAC; b6 = β-ACTIN 8505C 100 μm FAC.

## Data Availability

The datasets used and/or analysed in this study are reported within the manuscript and/or additional files and are available from the corresponding authors.

## Abbreviations

ATC: Anaplastic thyroid cancer
FAC: Ferric ammonium citrate
FTC: Follicular thyroid cancer
GPX4: Glutathione peroxidase 4
LOH: Lipid alcohols
LOOH: Lipid hydroperoxides
PTC: Papillary thyroid cancer
RSL3: RAS-selective lethal 3
SLC7A11: Solute carrier family 7 member 11
TfR1: Type I transferrin receptor
