# Supplementary material for: Anaplastic thyroid cancer cells reduce CD71 levels to increase iron overload tolerance
Source: J Transl Med. 2023 Nov 3;21:780. doi: 10.1186/s12967-023-04664-9 (PMC10625232; doi:10.1186/s12967-023-04664-9)
Supplement: Supplementary file 1 — Additional file 1: Table S1. Descriptive statistics for the S.F. (% over CTRL) observed in FTC-133 and 8505C cells, after treatment with 0, 50, 100 μM of FAC. S.F., surviving fraction; CTRL, control; FAC, ferric ammonium citrate. Figure S1. Original, uncropped western blot images showed in Figure 8 for FTC-133 (a) and for 8505C (b). Samples are indicated as a1 = CD71 FTC-133 CTRL; a2 = CD71 FTC-133 50 μm FAC; a3 = CD71 FTC-133 100 μm FAC; a4 = β-ACTIN FTC-133 CTRL; a5 = β-ACTIN FTC-133 50 μm FAC; a6 = β-ACTIN FTC-133 100 μm FAC; b1 = CD71 8505C CTRL; b2 = CD71 8505C 50 μm FAC; b3 = CD71 8505C 100 μm FAC; b4 = β-ACTIN 8505C CTRL; b5 = β-ACTIN 8505C 50 μm FAC; b6 = β-ACTIN 8505C 100 μm FAC. [file 12967_2023_4664_MOESM1_ESM.pdf]

	FTC-133			8505C		
	**CTRL**	**50 µm FAC**	**100 µm FAC**	**CTRL**	**50 µm FAC**	**100 µm FAC**
**Mean**	100.00	42.68	24.24	100.00	13.66	3.36
**Std. Deviation**	41.70	11.06	10.96	7.57	4.02	1.37
**Std. Error of Mean**	17.02	4.52	4.47	3.39	1.80	0.61

Table S1. Descriptive statistics for the S.F. (% over CTRL) observed in FTC-133 and 8505C cells, after treatment with 0, 50, 100 µM of FAC. S.F., surviving fraction; CTRL, control; FAC, ferric ammonium citrate.

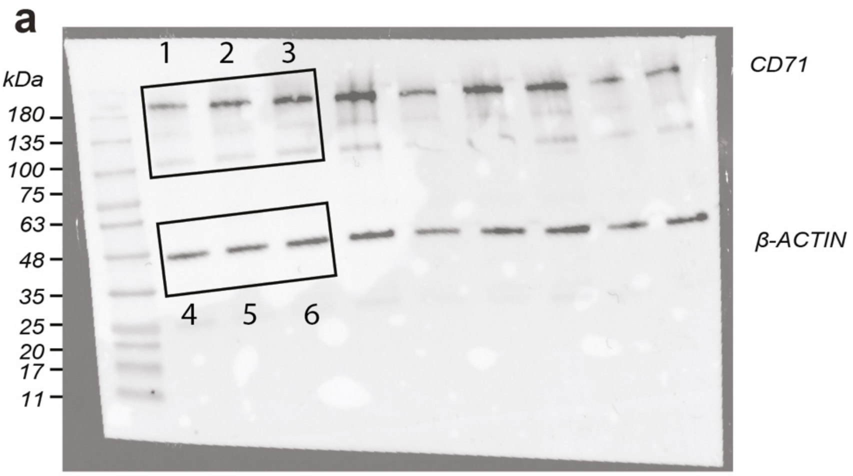

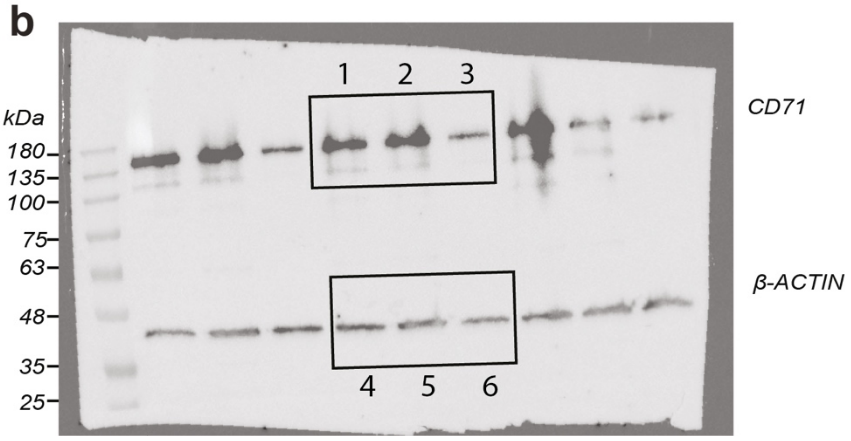

Fig. S1 Original, uncropped western blot images showed in Figure 8 for FTC-133 (a) and for 8505C (b). Samples are indicated as a1 = CD71 FTC-133 CTRL; a2 = CD71 FTC-133 50 μm FAC; a3 = CD71 FTC-133 100 μm FAC; a4 = β-ACTIN FTC-133 CTRL; a5 = β-ACTIN FTC-133 50 μm FAC; a6 = β-ACTIN FTC-133 100 μm FAC; b1 = CD71 8505C CTRL; b2 = CD71 8505C 50 μm FAC; b3 = CD71 8505C 100 μm FAC; b4 = β-ACTIN 8505C CTRL; b5 = β-ACTIN 8505C 50 μm FAC; b6 = β-ACTIN 8505C 100 μm FAC.
